# Paraventricular thalamic nucleus plays a critical role in consolation and anxious behaviors of familiar observers exposed to surgery mice

**DOI:** 10.7150/thno.45690

**Published:** 2021-02-06

**Authors:** Qiuting Zeng, Weiran Shan, Hui Zhang, Jianjun Yang, Zhiyi Zuo

**Affiliations:** 1 Department of Anesthesiology, University of Virginia, Charlottesville, VA 22908, U.S.A.; 2 Department of Anesthesiology, Zhongda Hospital, School of Medicine, Southeast University, Nanjing, 210009, China.; 3 Department of Anesthesiology, Pain and Perioperative Medicine, The First Affiliated Hospital, Zhengzhou University, Zhengzhou, Henan, China.

**Keywords:** anxious behavior, consolation behavior, mice, orexin, paraventricular thalamic nucleus, surgery.

## Abstract

**Background:** Consolation behaviors toward the sick are common in humans. Anxiety in the relatives of the sick is also common. Anxiety can cause detrimental effects on multiple systems. However, our understanding on the neural mechanisms of these behaviors is limited because of the lack of small animal models.

**Methods:** Five of 6- to 8-week-old CD-1 male mice were housed in a cage. Among them, 2 mice had right common artery exposure (surgery) and the rest were without surgery. Allo-grooming and performance in light and dark box and elevated plus maze tests of the mice were determined.

**Results:** Mice without surgery had increased allo-grooming toward mice with surgery but decreased allo-grooming toward non-surgery intruders. This increased allo-grooming toward surgery mice was higher in familiar observers of surgery mice than that of mice that were not cage-mates of surgery mice before the surgery. Familiar observers developed anxious behavior after being with surgery mice. Surgery mice with familiar observers had less anxious behavior than surgery mice without interacting with familiar observers. Multiple brain regions including paraventricular thalamic nucleus (PVT) were activated in familiar observers. The activated cells in PVT contained orexin receptors. Injuring the neurons with ibotenic acid, antagonizing orexin signaling with an anti-orexin antibody or inhibiting neurons by chemogenetic approach in PVT abolished the consolation and anxious behaviors of familiar observers.

**Conclusions:** Mice show consolation behavior toward the sick. This behavior attenuates the anxious behavior of surgery mice. The orexin signaling in the PVT neurons play a critical role in the consolation of familiar observers toward surgery mice and their anxious behavior. Considering that about 50 million patients have surgery annually in the United States, our study represents the initial attempt to understand neural mechanisms for consolation and anxiety of a large number of people.

## Introduction

Consolation behaviors toward the sick are a common presentation in humans and an important component of empathetic responses. These behaviors include giving a hug or gentle touching to the sick in humans 1. About 50 million patients have surgery each year in the United States 2. Relatives, friends or even strangers often show consolation behavior toward these patients. Various forms of empathy, such as targeted helping and mirroring, have been shown in non-human primates and elephants, animals with advanced cognitive capacities 3. Empathy for pain and toward distressed others as well as contagious itch behavior have been shown in mice 4-6. The distress stimuli include pain induced by various etiologies and social defeat 6-9. However, small animal model of consolation behavior toward the sick has not been established and the neural mechanisms for this behavior are not known.

Patients and their relatives are often anxious when they have surgery. Anxiety is known to cause physiological responses and worsen outcome of patients with various diseases 10-12. It is known that caregiving spouses of patients with dementia have an increased chance to suffer from dementia 13. The mechanism for this phenomenon is not clear. However, factors, such as increased anxiety and similar living environment, may contribute to the development of dementia in the caregiving spouses 13, 14. These findings suggest that anxiety of the relatives of surgical patients can have significant adverse effects on the health of surgical patients' relatives. However, no animal model simulating this anxiety has been reported and the neural and molecular mechanisms for this behavior have not been explored.

This study was aimed at determining whether small animals, such as mice, had consolation behavior toward surgical mice and developed anxious behavior after interacting with surgical mice and defining neural mechanisms for these behaviors.

## Methods and Materials

The animal protocol was approved by the Institutional Animal Care and Use Committee of the University of Virginia (Charlottesville, VA, USA). All animal experiments were carried out in accordance with the National Institutes of Health Guide for the Care and Use of Laboratory Animals (NIH publications number 80-23) revised in 2011 and reported according to the ARRIVE guidelines.

### Animals and animal groups

Six- to 8-week old CD-1 male mice (weighing 31 - 36 g) from Charles River Laboratories International Inc. (Wilmington, MA, USA) were used because this age of mice had consistent learning and memory impairment with 100% survival rate after surgery in our previous study 15. The mice were housed in cages (5 mice/cage) on a 12-h light/dark cycle with free access to water and food. All experimental procedures or behavior tests were conducted during the light phase.

**Figure 1:**
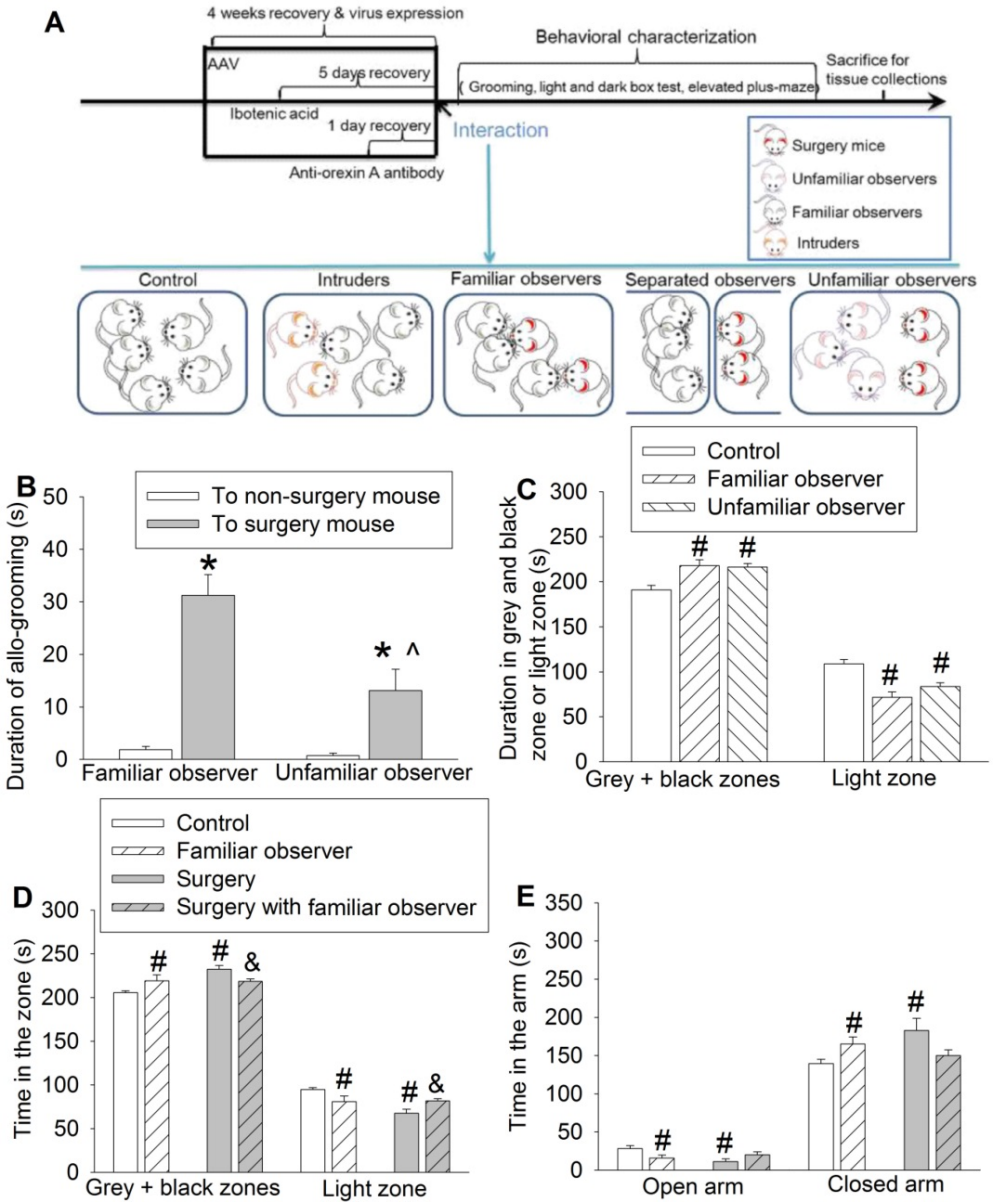
** Interaction with surgery mice induced consolation and anxious behavior in mice.** Familiar observers or unfamiliar observers were housed in the same cage with surgery mice for 1 h. **(A)** Diagram of experimental design. **(B)** Allo-grooming to mice without surgery and mice with surgery in the same cage in the first 10 min after they were placed together. **(C)** Performance of familiar observers and unfamiliar mice in the light and dark box. **(D)** Performance of familiar observers and surgery mice in the light and dark box test. **(E)** Performance of familiar observers and surgery mice in the elevated plus maze test. Results are mean ± SEM (n = 9 for unfamiliar observers and 16 for familiar observers in panels B and C, 7 - 11 for panel D, 9 - 12 for panel E). * P < 0.05 compared with the time of allo-grooming to non-surgery mice; ^ P < 0.05 compared with the corresponding values of familiar observers; # P < 0.05 compared with control; & P < 0.05 compared with surgery mice.

In the first experiment (Figure 1A), three separate cohorts of mice were used. In the first cohort, mice were randomized by a SPSS-generated random number assignment to one of five groups: control, surgery, familiar observer, separated observer and unfamiliar observer. Control mice lived together with other control mice (5 mice per cage that was 26 x 15 x 12 cm) and were not exposed to surgery mice or unfamiliar intruders. Surgery mice had surgery under anesthesia with 1.8% isoflurane for 2 h. Familiar observers were cage-mates of surgery mice prior to their surgery for at least 2 weeks. Separated observers were cage-mates of surgery mice prior to their surgery for at least 2 weeks and then three separated observers were in their home transparent cage next to another transparent cage housing two cage-mates but with surgery. Unfamiliar observers were not cage-mates of mice with surgery prior to surgery but were placed together with surgery mice after the surgery. Three non-surgery mice (either familiar observers or unfamiliar observers) were housed with 2 surgery mice. These mice were used for behavioral tests (allo-grooming, light and dark box or elevated plus maze tests). Separated observers were not subjected to these behavior tests because they were not in the same cage with surgery mice in the first hour after the surgery and, thus, the allo-grooming toward surgery mice could not be assessed. The second cohort mice, also in the five groups, were used to obtain the serum, hippocampus and prefrontal cortex for ELISA analyses of interleukin (IL)-6 at 2 h, 6 h and 24 h after one-hour interaction with surgery mice. Each mouse provided samples (blood and brain tissues) for one time point. The third cohort mice, also in the five groups, were used for harvesting the whole brain immediately after the one-hour interaction for c-Fos and NeuN immunofluorescent staining.

In the second experiment, brain of control mice, familiar observers and mice with surgery was harvested immediately after the one-hour interaction between familiar observers and surgery mice. These brains were used to define the heat map of c-Fos positive cells in the brain.

In the third experiment, 2 mice (intruder) from different cages were put into a cage with three resident mice. After one-hour interaction when allo-grooming behavior was assessed, they were tested in light and dark box and elevated plus maze tests.

In the fourth experiment, 5 control mice were injected with pAAV2-hsyn-EGFP (Table S1) into the paraventricular thalamic nucleus (PVT) region. Their brains were harvested to determine the PVT projections 4 weeks later. Brains from 8 familiar observers were used to determine whether c-Fos positive cells expressed orexin receptors.

In the fifth experiment, mice were randomly assigned to four groups: control, familiar observers, familiar observers plus ibotenic acid and familiar observers plus vehicle. Familiar observers received injection of 300 nl 0.3% ibotenic acid or phosphate buffered saline (PBS, vehicle) into PVT. Ibotenic acid was used to destroy neurons in PVT. They were allowed for recovery for 5 days and then interacted freely with surgery mice for 1 h. These mice were used for behavioral tests (allo-grooming, allo-licking and light and dark box tests). After the behavioral tests, their brains were harvested to examine neuronal injury in PVT.

In the sixth experiment, mice were randomly assigned to five groups: control, familiar observers, familiar observers plus hM4Di plus compound-21, familiar observers plus hM4Di plus vehicle, and familiar observers plus mCherry plus compound-21 (Table S1). Mice were injected with pAAV2-hsyn-hM4Di-mCherry or pAAV2-hsyn-mCherry into PVT. Four weeks later, they were injected intraperitoneally with a designer receptors exclusively activated by designer drug (DREADD) agonist (compound 21) at 3 mg/kg in 90 µl or 90 µl 0.2% DMSO in normal saline (vehicle), solvent for compound 21, 30 min before the one-hour interaction between familiar observers and mice with surgery. The combination of hM4Di and compound 21 was used to inhibit neurons. Compound 21 was used because it has good penetration into the brain and does not generate active metabolite while clozapine-N-oxide, another DREADD agonist, generates the active metabolite clozapine 16. The mice were used for behavioral tests (allo-grooming, allo-licking, light and dark box tests). After the behavioral tests, the mice brains were harvested to examine the location of mCherry expression and c-Fos expression in PVT.

In the seventh experiment, mice were randomly assigned to four groups: control, familiar observers, familiar observers plus an anti-orexin A antibody, familiar observers plus heat-inactivated orexin A antibody. Familiar observers were injected with an anti-orexin A antibody or heat-inactivated anti-orexin A antibody into PVT one day before the one-hour interaction between familiar observers and mice with surgery. The anti-orexin antibody was used to bind and block orexin signaling in the PVT neurons. The mice were used for behavioral tests (allo-grooming, allo-licking and light and dark box tests).

### Animal surgery

The surgery was right carotid arterial exposure 15, 17. Briefly, mice were anesthetized by 1.8% isoflurane that was delivered by an isoflurane vaporizer. A 1.5-cm midline neck incision was made after the mouse was exposed to isoflurane at least for 30 min. The soft tissues over the trachea were retracted gently. One centimeter long right common carotid artery was dissected carefully free from adjacent tissues without damaging the vagus nerve. The wound was then irrigated and closed by using surgical sutures. The surgical procedure was performed under sterile conditions and lasted about 15 min. After the surgery, all animals received a subcutaneous injection of 3 mg/kg bupivacaine. The total duration of anesthesia was 2 h, a clinically relevant duration of anesthesia. No response to toe pinching was observed during the whole course of anesthesia. During anesthesia, rectal temperature was monitored and maintained at 37 °C with the aid of servo-controlled warming blanket (TCAT-2LV, Physitemp instruments, Clifton, NJ). Control mice, familiar observers, unfamiliar observers and separated observers did not receive anesthesia, surgery or bupivacaine. The pain levels of mice with or without surgery during their 1 h interaction for assessing allo-grooming behavior was scored as described previously based on orbital tightening, nose bulge, cheek bulge, ear position and whisker change with the maximal score of 20 18.

### Injection of virus or chemicals into paraventricular thalamic nucleus

Viruses or chemicals were injected into PVT at 3 sites (100 nl for each site) along anterior-posterior axis with coordinates at -1.0, -1.30 and -1.60 mm, respectively, from Bregma, 0.05 mm from midline and 3 mm in depth. Viruses were pAAV2-hsyn-hM4Di-mCherry, pAAV2-hsyn-mCherry or pAAV2-hSyn-EGFP (Table S1). The mice were used in experiments 4 weeks after the injection. Chemicals were 0.3% ibotenic acid, an anti-orexin A antibody and its heat-inactivated anti-orexin A antibody (Table S1). Mice were used in experiments 5 days after ibotenic acid injection or 1 day after antibody injection as described previously 19-21. These times were allowed for ibotenic acid to destroy neurons and antibody to diffuse and bind orexin receptors in PVT.

### Allo-grooming time

Allo-grooming was defined as head contact with the body or head of the surgery individual, accompanied by a rhythmic head movement 4, while allo-licking was defined as head contact with the surgical incision of the surgery individual, accompanied by a rhythmic head movement 4. Grooming directed toward the rear (genitals, anogenital region, or tail) was excluded. The allo-licking time was separated from allo-grooming time in experiment 5 to experiment 7 to determine the neural mechanisms for regulating allo-licking. The allo-grooming time in the other experiments included allo-licking time. For assessment of mouse behavior, investigators were blinded to the group assignment of mice.

### Light and dark box test

The light and dark box was made of white and black opaque Plexiglas (19 cm width × 19 cm length × 25 cm height light chamber and dark chamber). These two chambers were connected by a central grey corridor (6.5 cm width × 9 cm length × 25 cm height). The light chamber was brightly illuminated by white light and the other was dark. Mice were placed in the middle of the light chamber facing a side away from the door and then released. Behavior was recorded for 5 min. The time spent in each chamber was recorded. After each trial, the apparatus was cleaned with 70% ethanol.

### Elevated plus-maze test

As previous described 22, mice were placed in the central area of the maze. As they freely explored the maze, their behavior was recorded for 5 min by a video camera mounted above the maze and analyzed using ANY-maze system (SD Instruments). The duration in open arms or closed arms was calculated to measure anxiety-like behavior.

### Blood and brain tissue harvest

Mice were deeply anesthetized with isoflurane for 2 min. Blood was harvested at 2 h, 6 h or 24 h after the one-hour interaction of mice with surgery mice and centrifuged at 1300 g for 20 min at 4 °C after it had been placed at 4 °C for 2 h for serum collection. All mice for brain tissue harvesting were first perfused transcardially with normal saline. Various parts of the brain were then dissected depending on the experiments. The hippocampus and prefrontal cortex were dissected out immediately for ELISA assay. The cerebral hemisphere from Bregma -1.1 to -1.7 mm was used for immunofluorescent staining of PVT. The whole brain was used to determine c-Fos expression heat map.

### ELISA of IL-6

Since IL-6 is increased in the brain after surgery 15 and is the major cytokine induced by stress to mediate immunometabolic response 23, IL-6 in the serum, hippocampus and prefrontal cortex was measured by using ELISA kits (Table S1). Hippocampus and prefrontal cortex were homogenized on ice in the RIPA buffer (25 mM Tris-HCl with pH 7.6, 150 mM NaCl, 1% sodium deoxycholate, and 0.1% SDS) (Thermo Scientific, Rockford, IL, USA) and a protease inhibitor cocktail (10 mg/ml aproteinin, 5 mg/ml peptastin, 5 mg/ml leupetin, and 1 mM phenylmethane sulfonylfluoride) (Sigma-Aldrich, St. Louis, MO, USA). After being centrifuged for 20 min (13,000 g, 4 °C), the supernatant was collected for ELISA detection according to the manufacturer's instruction. Cytokines in the serum were detected in the same way. The amount of IL-6 in each sample except for those in the serum was standardized by its protein content. The results from brain samples of animals under various experimental conditions were then normalized by the mean values of the corresponding control animals in each ELISA assay.

### Immunofluorescent staining

Immunofluorescent labeling was performed as we did before 24 to detect neuronal nuclear (NeuN), c-Fos and orexin receptors 1 and 2. In brief, mice were sacrificed under deep anesthesia and transcardially perfused with saline. Brains were harvested and post-fixed in 4% paraformaldehyde in 0.1 M PBS at 4 °C for 24 h, dehydrated, and embedded in paraffin. Coronal 5-μm sections from -1.1 to -1.7 mm (for PVT), 1.34 to 1.70 mm (for insular cortex) or -4.96 to -5.02 mm (for parabrachial nucleus) relative to bregma were cut sequentially and mounted on microscope slides. Antigen retrieval with sodium citrate buffer (10 mM sodium citrate, 0.05% Tween 20, pH 6.0) was performed at 95 - 100 °C for 20 min. After being washed in Tris-buffered saline (TBS) containing 0.1% triton-X 100, sections were blocked in 10% donkey serum plus 1% bovine serum albumin in TBS for 2 h at room temperature and then incubated at 4 °C overnight with the following primary antibodies: mouse monoclonal anti-NeuN (1:200 dilution), rabbit polyclonal anti-c-Fos (1:1000 dilution), mouse monoclonal [2H2] anti-c-Fos (1:1000 dilution; used in the double staining of c-Fos and orexin receptors) and rabbit polyclonal anti-orexin receptor 1 and 2 antibody (1:100 dilution) (Table S1). Sections were rinsed in TBS with 0.1% triton-X 100 and then incubated with donkey anti-mouse IgG antibody conjugated with Alexa Fluor 488 (1:200 dilution), donkey anti-rabbit IgG antibody conjugated with Alexa Fluor 488 (1:200 dilution), donkey anti-mouse IgG antibody conjugated with Alexa Fluor 594 (1:200 dilution) or donkey anti-rabbit IgG antibody conjugated with Alexa Fluor 594 (1:200 dilution) (Table S1) for 1 h at room temperature in the dark. The slides were then rinsed in TBS three times for 5 min each, stained with 4',6-diamidino-2-phenylindole (DAPI) and cover-slipped with VECTASHIELD HardSet mounting medium. For quantification of the number of c-Fos positive cells in each microscopic field, three randomly selected and independent microscopic fields in the PVT areas were acquired in each brain section. Three sections from each mouse were analyzed. The 9 values from each mouse were averaged to reflect c-Fos expression in that mouse. The examiner was blind to the group assignment of the brain sections.

Frozen coronal sections (25 μm) from -1.1 to -1.7 mm relative to bregma were used to examine the location of mCherry expression. The slides were rinsed in TBS three times for 5 min each, stained with DAPI and cover-slipped with VECTASHIELD HardSet mounting medium.

For the tracing experiment, 25 µm thick frozen coronal brain sections were cut sequentially from the whole brain in an anterior to posterior direction. One section was kept from every 10 sections to examine the expression of EGFP whose code was carried by pAAV2-hSyn-EGFP injected into PVT.

### Heat map of regions with c-Fos expression

As previously reported 5, frozen coronal brain sections of 25 µm thickness were cut sequentially from the whole brain in an anterior to posterior direction. One section was kept from every 10 sections for staining c-Fos and DAPI. Images from brain sections of the c-Fos positively stained regions were collected with a confocal microscope system. Three randomly selected and independent microscopic fields in each brain structure of interest were acquired from each brain section. Three sections from each mouse were analyzed by image J to calculate the mean value of the number of c-Fos positive cells per microscopic field for the mouse. The 9 values from each mouse were averaged to reflect c-Fos expression in the brain region of that mouse. The examiner was blind to the group assignment of the brain sections. Identification of brain anatomical regions was based on the morphology and DAPI counter staining images.

### Statistical analysis

Results in normal distribution are presented as means ± SEM (n ≥ 5) in figures. Data in non-normal distribution are in box plots (n ≥ 5). Data in figure 1B were analyzed by two-way analysis of variance (familiar observers and to surgery mice as the two factors). The comparison between control and familiar observers or control and surgery mice in the heat map results was performed by t-test. Comparison of pain scores was performed by rank sum test. The other data were analyzed by one-way analysis of variance followed by Tukey's test with normally distributed data or by one-way analysis of variance on ranks followed by the Tukey test with non-normally distributed data. Differences were considered significant at a P < 0.05. All statistical analyses were performed with SigmaStat (Systat Software, Inc., Point Richmond, CA, USA).

## Results

### Cage-mates of mice with surgery developed consolation and anxious behaviors

To address our study arms, we subjected adult CD-1 male mice to right common carotid artery exposure (surgery). Each cage contained 2 mice with this surgery and 3 mice without surgery as we did previously 25. The pain score was 7 (5, 8.5) [medium (25^th^ - 75^th^)] (n = 12) for these surgery mice during the 1 h interaction with non-surgery mice and 8 (6, 8.75) (n = 11, P = 0.505 for comparison) for surgery mice without interaction with non-surgery mice. The pain score of all non-surgery mice including familiar observers, controls or residents was 0. These results suggest that these non-surgery mice do not have spontaneous pain expression, which is different from increased mechanical pain sensitivity that can be contagious 9. We did not observe fighting behavior among the mice during the one-hour interaction. The duration of allo-grooming behavior in 10 min immediately after they were placed together in the cage was counted. Familiar observers or unfamiliar observers to the surgery mice had increased allo-grooming toward surgery mice compared with that toward other non-surgery mice (Figure 1B). This allo-grooming toward surgery mice was more in the familiar observers than that in the unfamiliar observers [F(1,46) = 7.543, P = 0.009]. These results suggest consolation behavior of non-surgery mice toward surgery mice. Familiarity increases this behavior. Both familiar observers and unfamiliar observers after being placed with the surgery mice for 1 h spent more time in the grey zone and black zone and less time in the light zone of a light and dark box than control mice (Figure 1C, Table S2), suggesting that familiar observers and unfamiliar observers develop anxious behavior after interacting with surgery mice. In another experiment, familiar observers after being exposed to surgery mice spent a longer time in the grey and black zones and a shorter time in the light zone than control mice (Figure 1D), which reproduced the same findings in the previous experiment. These mice also spent less time in the open arm and more time in the closed arm of elevated plus maze (Figure 1E), another indication of anxious behavior in these mice. Similar to the familiar observers, mice with surgery spent more time in the grey and black zones and less time in the light zone of light and dark box test and more time in the closed arm and less time in the open arm of elevated plus maze test than the control mice, suggesting that surgery mice have anxious behavior (Figures 1D-E). However, the surgery mice that had stayed with familiar observers for one hour spent more time in the light zone and less time in the grey and black zones than the surgery mice without interaction with familiar observers (Figure 1D). These results suggest that surgery mice with familiar observers are less anxious than surgery mice without interaction with non-surgery mice.

**Figure 2:**
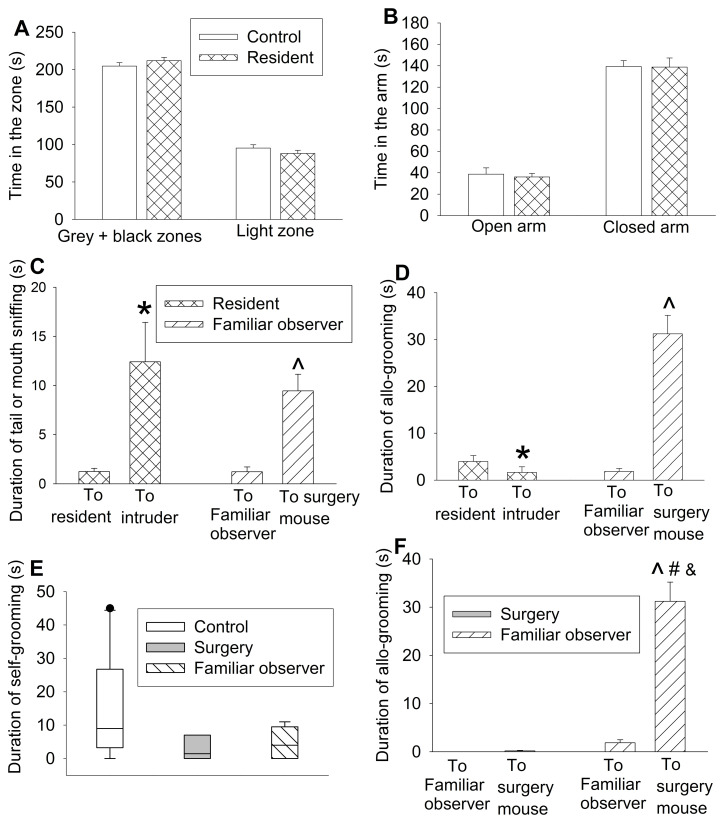
** Interaction with surgery mice but not with intruders induced consolation in mice.** Familiar observers were housed in the same cage with surgery mice for 1 h or intruders were housed in the same cage with original resident mice for 1 h. **(A)** Performance in the light and dark box test. **(B)** Performance in the elevated plus maze test. **(C)** Tail or mouth sniffing behavior. **(D)** Allo-grooming behavior of familiar observers and resident mice. **(E)** Self-grooming behavior. **(F)** Allo-grooming behavior of surgery mice and familiar observers. All results except for results in panel E are mean ± SEM (n = 12 - 14 for panel A, 6 - 10 for panel B, 9 - 12 for panel C, 16 - 18 for panel D, 12 - 16 for panel F). Results in panel E are in box plot format (n = 9 - 12). ● : lowest or highest score (the score will not show up if it falls in the 95th percentile); between lines: 95th percentile of the data; inside boxes: 25th to 75th percentile including the median of the data. * P < 0.05 compared with corresponding values to resident mice; ^ P < 0.05 compared with the corresponding values to familiar observers; # P < 0.05 compared with the allo-grooming value of surgery mice toward familiar observers; & P < 0.05 compared with the allo-grooming value of surgery mice toward surgery mice.

To determine whether the allo-grooming and anxious behaviors of the familiar observers were specific to their interaction with surgery mice, 2 intruder mice were introduced into a cage with 3 resident mice. There was no difference between resident mice and control mice in the amount of time in the grey and black zones and light zone of light and dark box and open and closed arms of elevated plus maze (Figures 2A-B). Although resident mice had an increased tail or mouth sniffing behavior toward intruders, the allo-grooming of residents toward intruders was decreased. However, familiar observers had increased tail or mouth sniffing and allo-grooming toward surgery mice (Figures 2C-D). There was no difference among control mice, surgery mice and familiar observers in self-grooming (Figure 2E, Table S2). Surgery mice had very little allo-grooming toward familiar observers or other surgery mice. However, familiar observers had more allo-grooming toward surgery mice than that toward other familiar observers (Figure 2F). These results suggest that the allo-grooming of familiar observers toward surgery mice was specific and that the increased anxious behavior of familiar observers after being exposed to surgery mice was also specific.

### Multiple brain regions including PVT were activated in surgical mice and their non-surgery cage-mates

**Figure 3:**
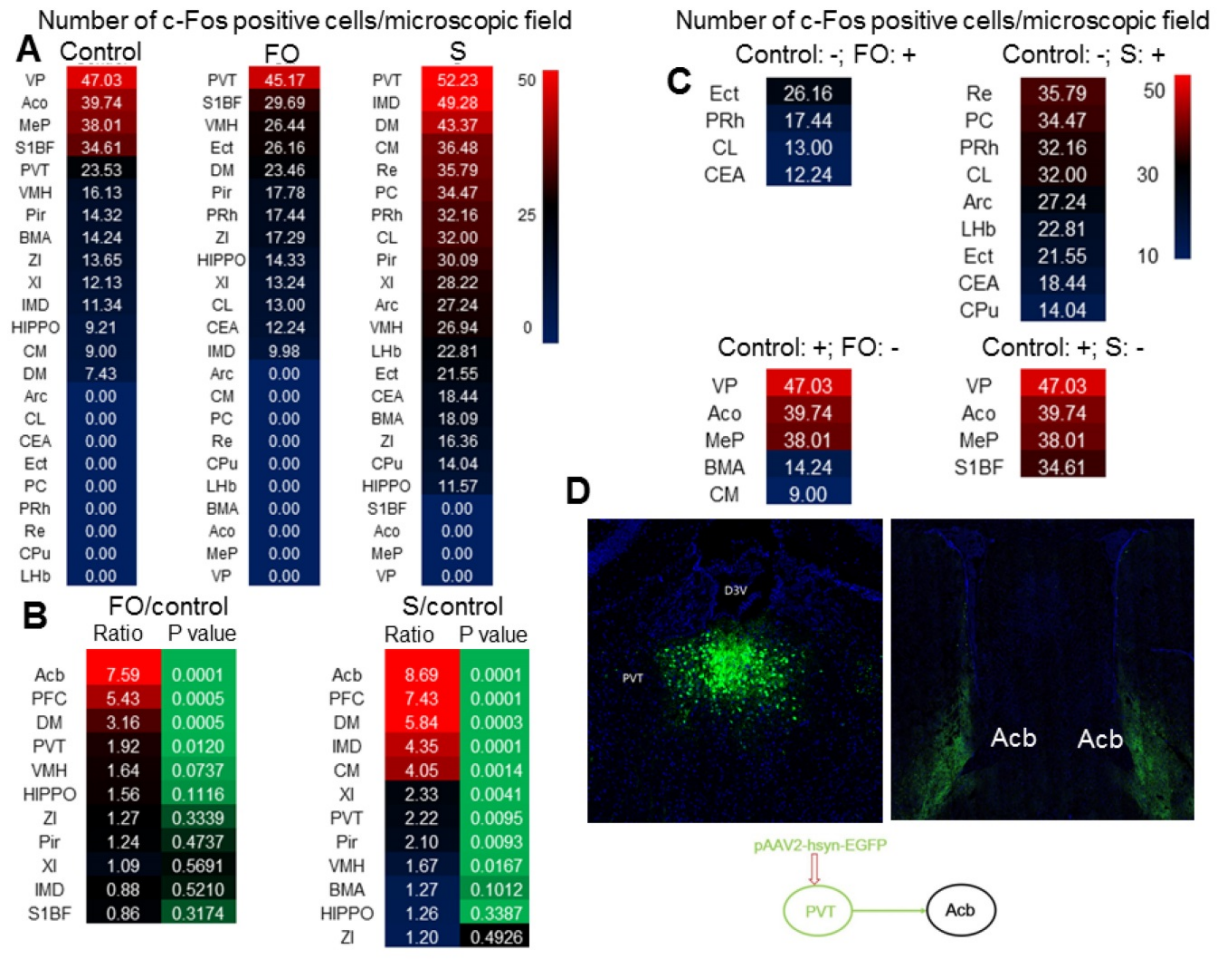
** Surgery and interaction of familiar observers with surgery mice induced changes in c-Fos expression.** The interaction was for 1 h and the brain was harvested for immunofluorescent staining. **(A)** Heat map of the number of c-Fos positive cells in control, familiar observers and surgery mice. **(B)** Heat map of the comparisons between familiar observers and control mice or surgery mice and control mice. **(C)** Heat map showing special cases of c-Fos positive cell patterns. Up two panels: no c-Fos positive cells in control mice. Bottom two panels: no c-Fos positive cells in familiar observers or surgery mice. -: no c-Fos positive cells. +: with c-Fos positive cells. **(D)** Tracing of PVT to Acb by injection of viral vector containing code for EGFP to PVT. Results are mean (n = 5 - 6). FO: familiar observers; S: surgery. Abbreviations for brain regions: Acb: nucleus accumbens; Aco: anterior cortical amygdaloid nucleus; Arc: arcuate hypothalamic nucleus; BMA: anterior part of basomedial amygdaloid nucleus; CEA: central nucleus of the amygdala; CL: centrolateral thalamic nucleus; CM: central medial thalamic nucleus; CPu: caudate putamen (striatum); D3V: third ventricle; DM: dorsomedial hypothalamic nucleus; ECT: ectorhinal cortex; HIPPO: hippocampus; IMD: intermediodorsal thalamic nucleus; MeP: posterodorsal part of medial amygdaloid nucleus; LHb: lateral habenular nucleus; PC: paracentral thalamic nucleus; PFC: prefrontal cortex; Pir: piriform cortex; PRh: perirhinal cortex; VP: ventral posterolateral thalamic nucleus; PVT: paraventricular thalamic nucleus; Re: reuniens thalamic nucleus; S1BF: barrel field of primary somatosensory cortex; VMH: ventromedial hypothalamic nucleus; XI: xiphoid thalamic nucleus; ZI: zona incerta.

As the first step to understand why familiar observers have consolation and anxious behaviors, c-Fos staining was used to identify the activated brain regions in familiar observers and surgery mice after they were placed together for 1 h after the surgery. PVT had the highest number of c-Fos positive cells among all brain regions in familiar observers and surgery mice (Figure 3A). There were more c-Fos positive cells in PVT of familiar observers and surgery mice than control mice. The number of c-Fos positive cells was increased in multiple other brain regions, such as nucleus accumbens (Acb) and prefrontal cortex (PFC), in the familiar observers and surgery mice and this increase was the highest in Acb and PFC when compared with control mice (Figure 3B). Interestingly, some brain regions, such as ectorhinal cortex (Ect), had c-Fos positive cells in familiar observers and surgery mice but not in control mice. On the other hand, c-Fos positive cells in some other brain regions, such as ventral posterolateral thalamic nucleus (VP), were found only in control mice but not in familiar observers and surgery mice (Figure 3C). Surgery mice had more brain regions with c-Fos positive staining than familiar observers (Figure S1). Neurons in PVT projected to Acb in our tracing experiment (Figure 3D) and to PFC 26. Since PVT is a major relay station to the limbic forebrain 26, is involved in learning, cue-rewarding processing and wakefulness 19, 27, 28 and had the highest c-Fos positive cells among all brain regions of the familiar observers, we focused to determine whether PVT played a role in the consolation and anxious behaviors of these mice.

**Figure 4:**
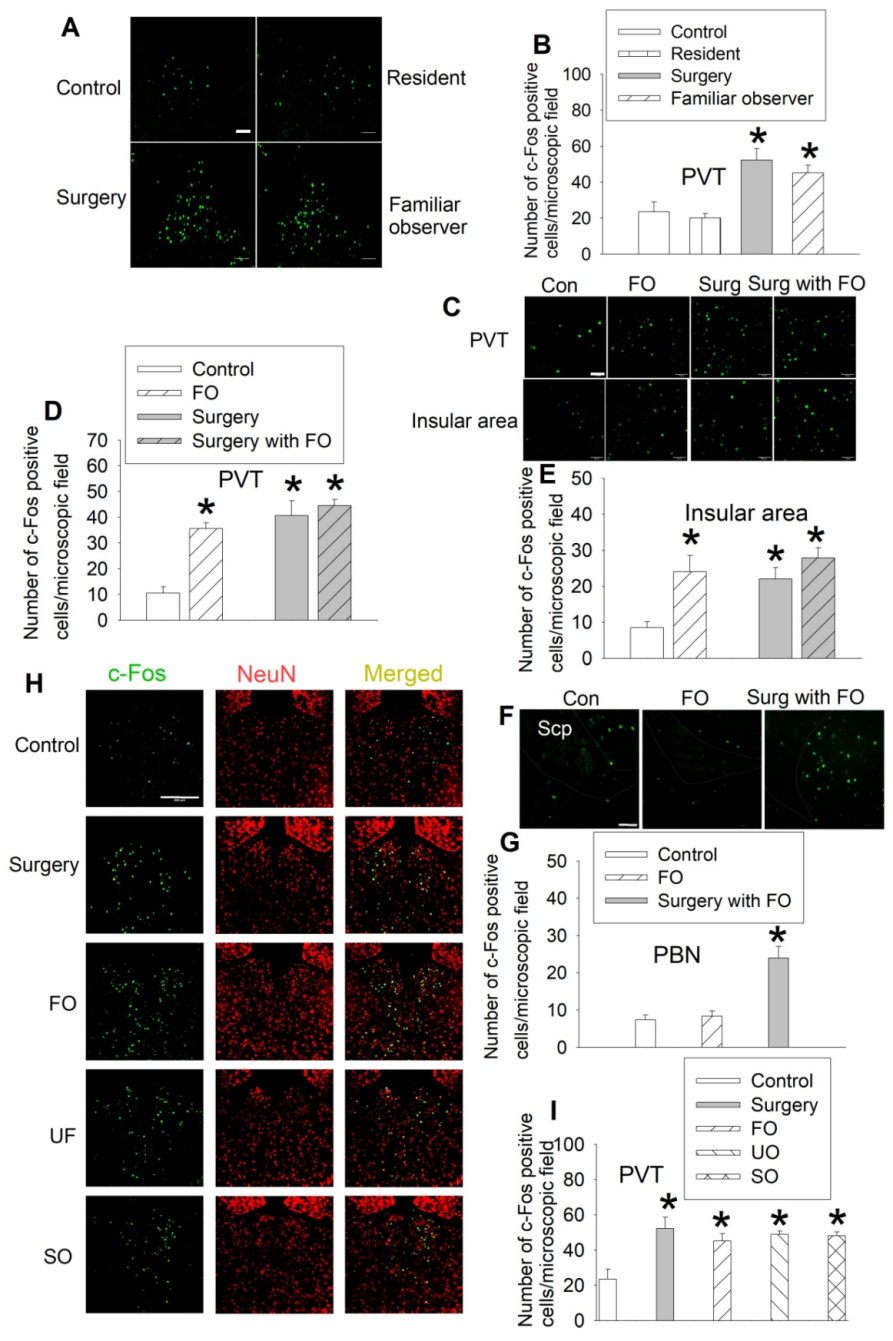
** Familiar observers but not resident mice had increased c-Fos positive cells in special brain regions.** Familiar observers or unfamiliar mice were housed in the same cage with surgery mice for 1 h or intruders were housed in the same cage with original resident mice for 1 h. The brain was harvested for immunofluorescent staining. **(A)** Representative images of c-Fos expression in PVT. Scale bar = 50 µm. **(B)** Quantification of c-Fos positive cells in PVT. **(C)** Representative images of c-Fos expression in PVT and insular cortex. Scale bar = 50 µm. **(D-E)** Quantification of c-Fos positive cells in PVT and insular cortex. **(F)** Representative images of c-Fos expression in the parabrachial nucleus. The edge of the nucleus is circulated. Scale bar = 100 µm. The inset in each panel is a high magnification microscopic field. **(G)** Quantification of c-Fos positive cells in the parabrachial nucleus. Scp: superior cerebellar peduncle. Scale bar = 50 µm. **(H)** Representative images of c-Fos expression in the PVT of various groups. Scale bar = 200 µm. **(I)** Quantification of c-Fos positive cells in the PVT of various groups. Results are mean ± SEM (n = 5 - 8). * P < 0.05 compared with control. Con: control; FO: familiar observer; PBN: parabrachial nucleus; PVT: paraventricular thalamic nucleus; SO: separated observer; Surg: surgery; UO: unfamiliar observer.

In a different set of mice, we reproduced the results that surgery mice and familiar observers had an increased number of c-Fos positive cells in their PVT. However, resident mice exposed to intruders did not have increased c-Fos positive cells in the PVT (Figures 4A-B, Table S2). Similarly, there were increased c-Fos positive cells in the PVT and insular cortex of familiar observers, surgery mice, and surgery mice after being interacted with familiar observers. There was no difference among these three types of mice in the number of c-Fos positive cells in these two brain regions (Figures 4C-E, Table S2). There was no difference between control mice and familiar observers but surgery mice after being interacted with familiar observers were higher than control and familiar observers in the number of c-Fos positive cells in the parabrachial nucleus (Figures 4F-G, Table S2), a hub for pain and aversion 29. These results suggest that familiar observers after being interacted with surgery have increased c-Fos positive cells in selected brain regions. Similarly, unfamiliar observers that stayed with surgery mice for 1 h had increased c-Fos positive cells in PVT. Separated observers that were familiar observers with surgery mice for at least 2 weeks before surgery mice had surgery and then stayed for 1 h in a cage next to the cage housing the surgery mice (Figure 1A) also had increased c-Fos positive cells in PVT (Figures 4H-I, Table S2). The cages housing the separated observers and surgery mice are transparent and mice in these two cages should be able to see each other. They might also be able to smell or hear each other because the cages were not air-tight. These results suggest that the activation of neurons in PVT does not require direct body contact and may be transferred by vision, smell or hearing. Thus, the activation of PVT may not be a result of performance of the allo-grooming.

### Cage-mates of surgical mice had increased proinflammatory cytokine in their brain

**Figure 5:**
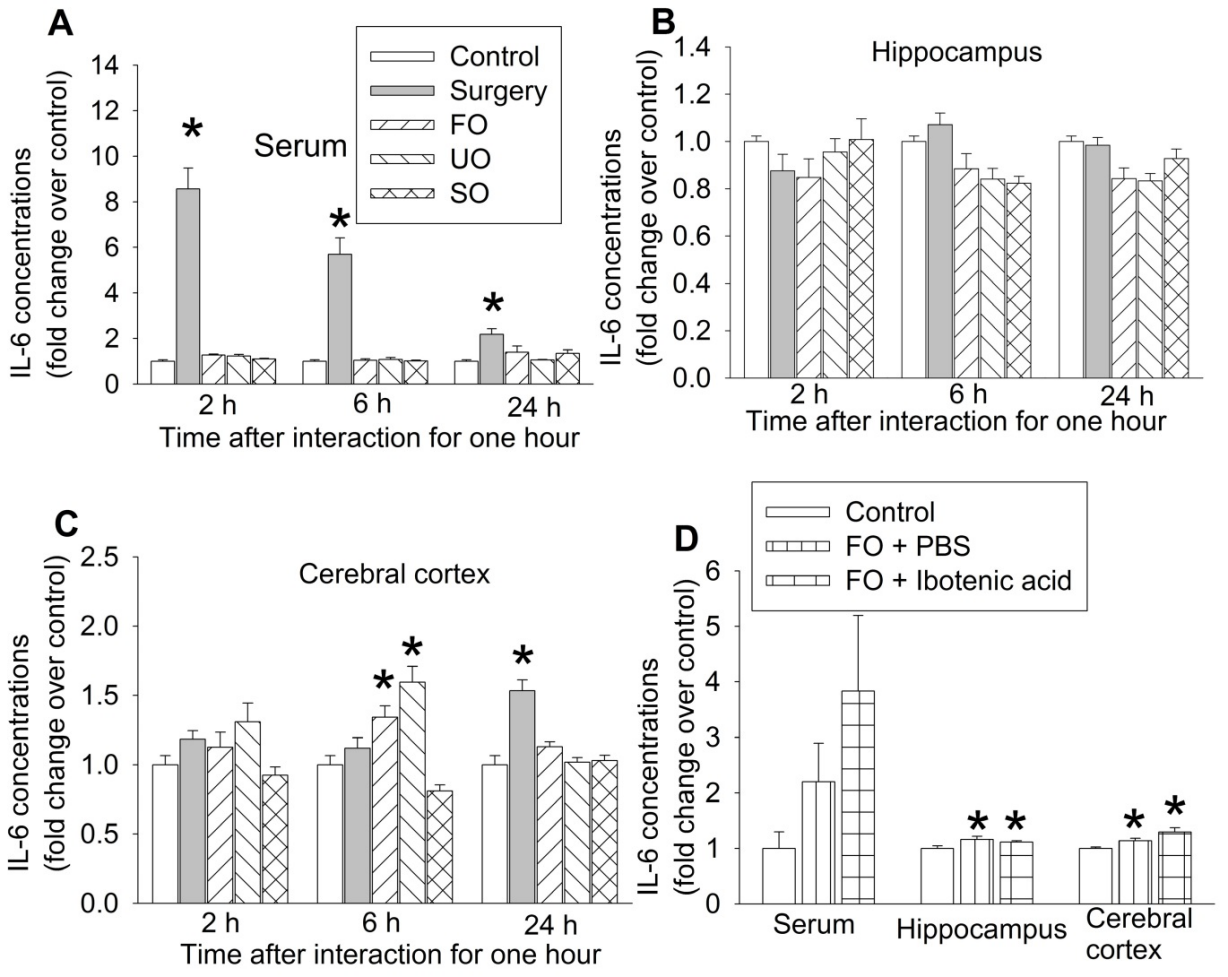
** The expression of interleukin-6 (IL-6) in blood and brain.** The interaction between surgery mice and others was for 1 h and the brain was harvested at various times after the interaction for results presented in panels A to C or at 6 h after the interaction for results presented in panel D. **(A)** IL-6 expression in the serum. **(B)** IL-6 expression in the hippocampus. **(C)** IL-6 expression in the frontal cortex. **(D)** IL-6 expression in the serum, hippocampus and frontal cortex. Results are mean ± SEM (n = 5 - 6 for panels A to C, 6 - 8 for panel D). * P < 0.05 compared with control. FO: familiar observer; PBS: phosphate buffered saline; SO: separated observer; UO: unfamiliar observer.

Our previous studies have shown that peripheral surgery increased proinflammatory cytokines, such as IL-6, in the blood and brain and that systemic inflammation induces neuroinflammation to impair learning and memory 15, 17, 24, 30. Consistent with those findings, surgery mice had increased IL-6 first in the blood and then in prefrontal cortex. The increase of IL-6 in prefrontal cortex occurred 24 h after the one-hour interaction in surgery mice. Although IL-6 was not increased in the blood and hippocampus of familiar observers, unfamiliar observers and separated observers within the 24-hour study period, IL-6 was increased in the prefrontal cortex of familiar observers and unfamiliar observers but not in the separated observers 6 h after the one-hour interaction with the surgery mice (Figures 5A-C, Table S2). These results suggest that increased proinflammatory cytokine in the brain does not require systemic inflammation in the familiar observers and unfamiliar observers. Since there was no increase in IL-6 in the separated observers (Figures 5A-C), the increase of IL-6 in the brain of familiar observers and unfamiliar observers may be induced by direct body interaction via multiple sensory pathways into the brain but may not be through vision. IL-6 increase in the prefrontal cortex of familiar observers and unfamiliar observers was earlier than that in surgery mice (Figure 5C), which suggests that the increase of IL-6 in the brain of familiar observers and unfamiliar observers may be via a route that is different from the surgery mice. Interestingly, the increased IL-6 in the hippocampus and cerebral cortex of familiar observers was not affected by ibotenic acid-induced PVT damage (Figure 5D, Table S2), suggesting that the increase of IL-6 in the brain is not controlled by PVT. PVT was not harvested for measuring IL-6 due to the concern whether PVT can be harvested accurately and PVT will provide enough samples for measuring IL-6 by ELISA.

### PVT and orexin receptor containing neurons were critical for consolation and anxious behaviors in cage-mates of surgical mice

**Figure 6:**
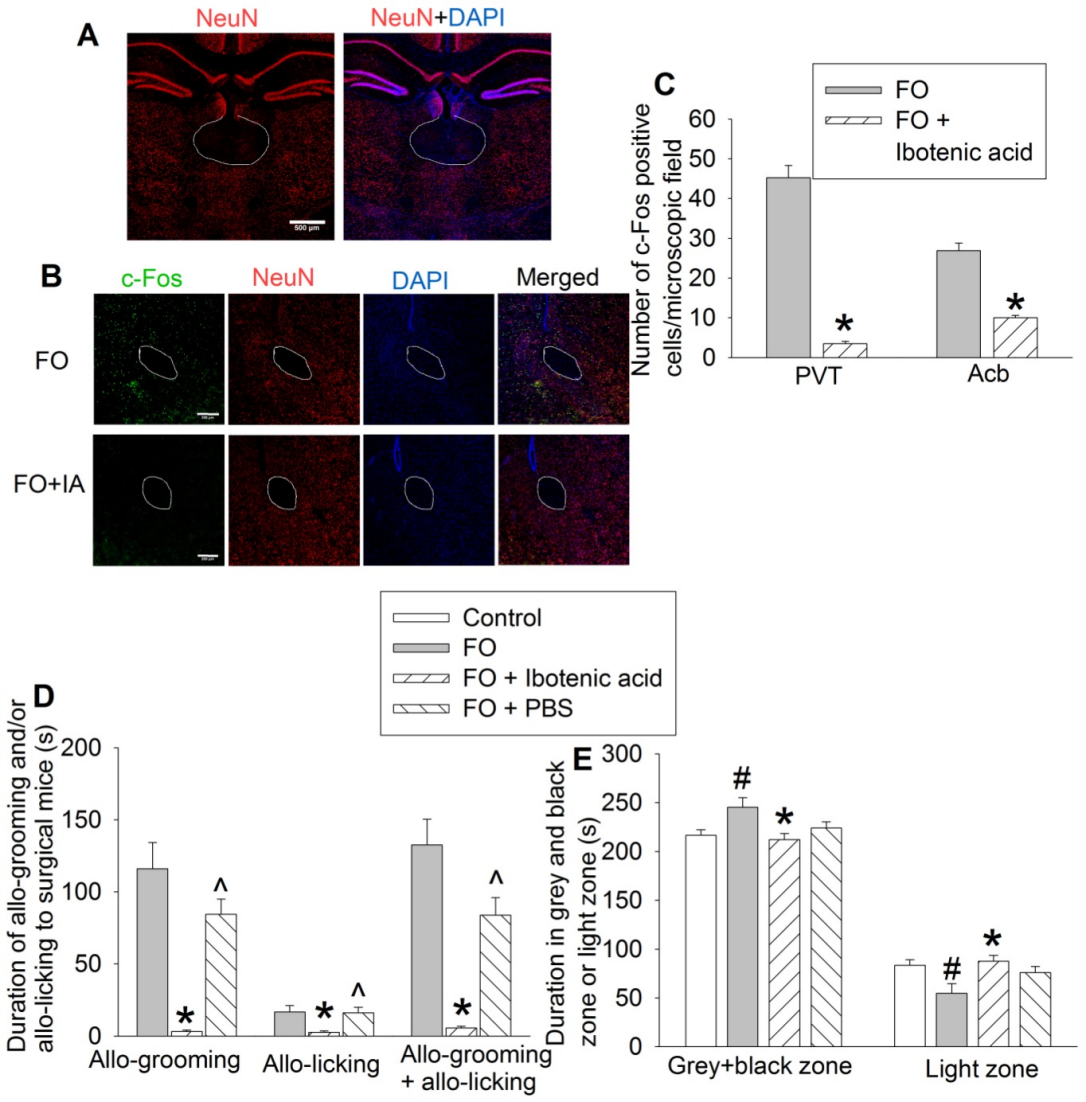
** Neuronal injury by ibotenic acid abolished consolation and anxious behavior.** Familiar observers received ibotenic acid injection into PVT and then used for experiments 5 days later. **(A)** Representatives of immunofluorescent staining showing neuronal damage in PVT. Scale bar = 500 µm. **(B)** Representatives of immunofluorescent staining showing that ibotenic acid-induced PVT neuronal injury decreased the expression of c-Fos positive cells in Acb. Scale bar = 200 µm. **(C)** Quantification of c-Fos positive cells in the PVT and Acb. **(D)** Allo-grooming and allo-licking of various groups of mice to mice with surgery in the same cage in the first 10 min after they were placed together. **(E)** Performance of mice in the light and dark box test. Results are mean ± SEM (n = 5 - 6 for panel C, 11 - 14 for panels D and E). * P < 0.05 compared with familiar observers; ^ P < 0.05 compared with familiar observers receiving ibotenic acid; # P < 0.05 compared with control. aca: anterior part of anterior commissure; FO: familiar observer; PBS: phosphate buffered saline; PVT: paraventricular thalamic nucleus.

To determine the role of PVT in the consolation and anxious behaviors of familiar observers, we used ibotenic acid, a neurotoxin 19, 31, to injure neurons in PVT. The majority of neurons in PVT were destroyed by ibotenic acid. This injury results in the block of activation of neurons in Acb that anatomically surround the anterior part of the anterior commissure (aca) in familiar observers (Figures 6A-C), suggesting that the activation of neurons in Acb of familiar observers is downstream of PVT. Mice with ibotenic acid injection to PVT did not show obvious changes in consciousness and motor functions during the behavioral testing. Injuring PVT neurons decreased the allo-grooming and allo-licking of familiar observers to the surgery mice (Figure 6D, Table S2) and reduced the time of familiar observers in the grey zone and black zone (Figure 6E, Table S2). Injection of PBS, a solvent for ibotenic acid, did not affect allo-grooming and allo-licking in the familiar observers (Figure 6D). These results suggest a critical role of PVT in the consolation of familiar observers toward surgery mice.

**Figure 7:**
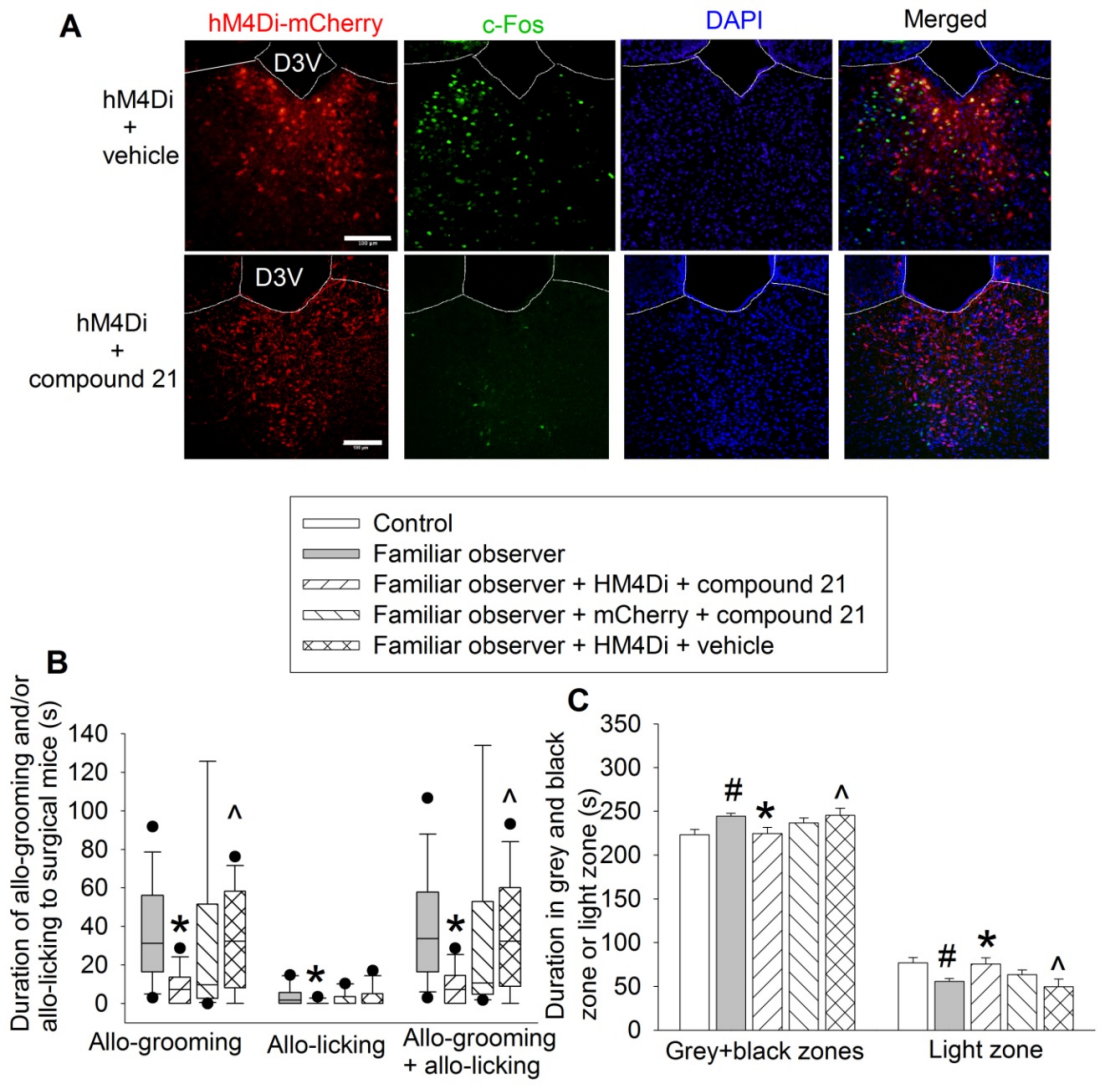
** Inhibition of PVT neurons abolished consolation and anxious behavior.** Familiar observers received injection of virus carrying code for hM4Di into PVT and then used for experiments 4 weeks later. **(A)** Representatives of immunofluorescent staining showing that chemogenetic approach inhibited the activation of PVT neurons. Scale bar = 100 µm. **(B)** Allo-grooming and allo-licking of various groups of mice to mice with surgery in the same cage in the first 10 min after they were placed together. **(C)** Performance of mice in the light and dark box test. Results in panel B are in box plot format (n = 11 - 15). ● : lowest or highest score (the score will not show up if it falls in the 95th percentile); between lines: 95th percentile of the data; inside boxes: 25th to 75th percentile including the median of the data. Results in panel C are mean ± SEM (n = 11 - 15). * P < 0.05 compared with familiar observers; ^ P < 0.05 compared with familiar observers receiving HM4Di and compound 21; # P < 0.05 compared with control. D3V: third ventricle.

Chemogenetic approach was used to test the findings from ibotenic acid experiments. Familiar observers received injection of pAAV2-hSyn-hM4D(Gi)-mCherry 4 weeks before they were interacting with surgery mice in the same cage (Figure 1A). The inhibition of neurons was induced by intraperitoneal injection of compound 21 (Figure 7A). Inhibiting PVT neurons decreased the allo-grooming and allo-licking of familiar observers to the surgery mice and reduced the time of familiar observers in the grey zone and black zone of the light and dark box. This decrease did not occur in familiar observers that received the viral injection but without compound 21 or injection of control virus plus compound 21 (Figures 7B-C, Table S2). In addition, familiar observers whose PVT neurons were inhibited had less allo-grooming and allo-licking to surgery mice and spent less time in the grey and black zones of the light and dark box than familiar observers that received viral and vehicle injection (Figures 7B-C). These results suggest that the activation of neurons in PVT is involved in the consolation and anxious behaviors of familiar observers.

**Figure 8:**
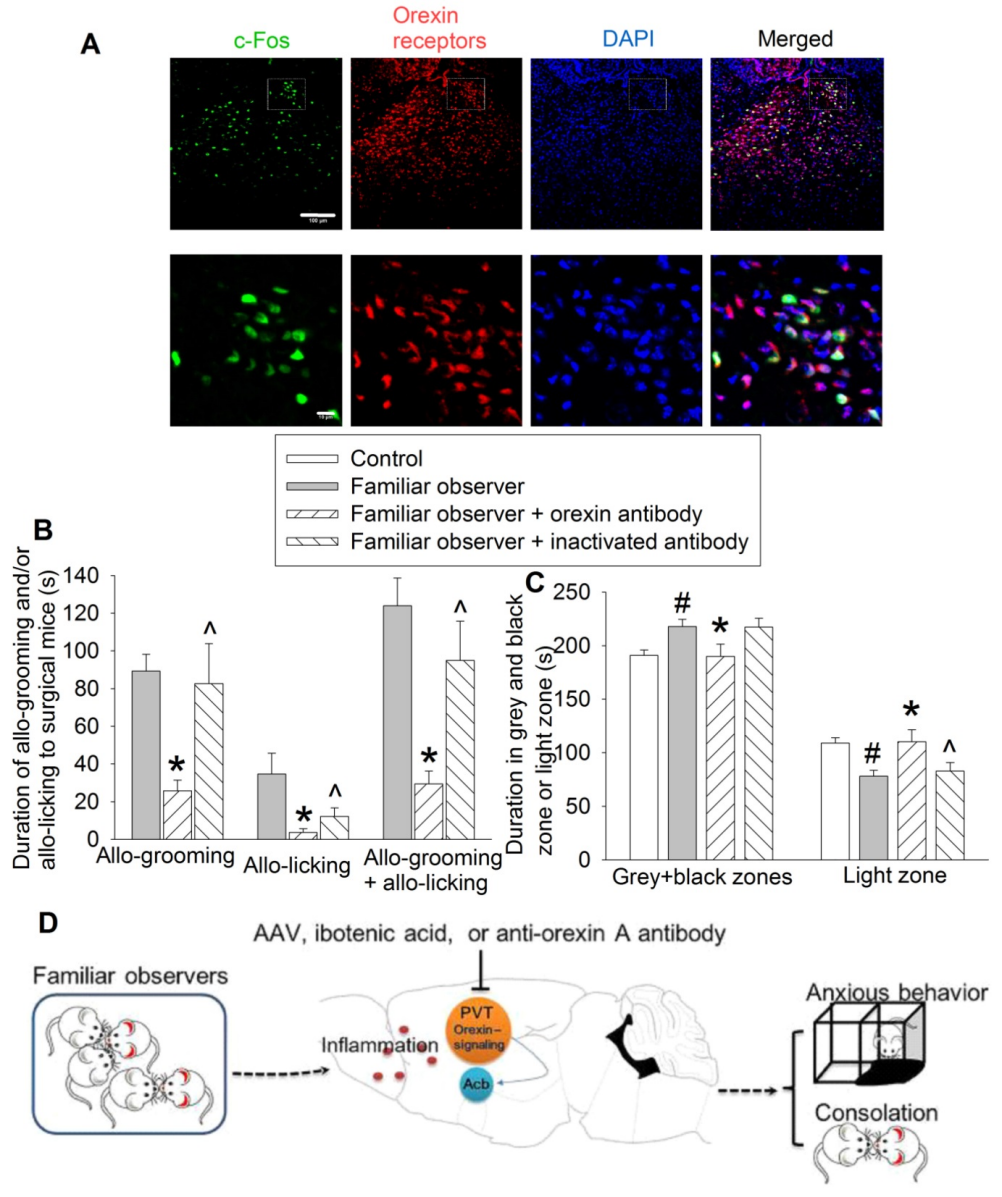
** Blocking orexin signaling in PVT abolished consolation and anxious behaviors.** Familiar observers received an anti-orexin antibody injection into PVT and were used for experiments one day later. **(A)** Representatives of immunofluorescent staining showing co-localization of orexin receptors with c-Fos in PVT. The images in the square of up panel are presented in the lower panel. Scale bar = 100 µm in up panel, = 10 µm in lower panel. **(B)** Allo-grooming and allo-licking of various groups of mice to mice with surgery in the same cage in the first 10 min after they were placed together. **(C)** Performance of mice in the light and dark box test. **(D)** Diagram presentation of our findings. Results are mean ± SEM (n = 10 - 15). * P < 0.05 compared with familiar observers; ^ P < 0.05 compared with familiar observers received an anti-orexin antibody; # P < 0.05 compared with control.

To determine the molecular mechanisms for the consolation and anxious behaviors of familiar observers, we first identified that almost all of c-Fos positive cells expressed orexin receptors (95 ± 2%, n = 8) (Figure 8A). Injection of an anti-orexin antibody into PVT decreased the allo-grooming and allo-licking of familiar observers to the surgery mice and reduced the time of familiar observers in the grey zone and black zone. This inhibition did not happen when heat-inactivated anti-orexin antibody was injected into PVT (Figures 8B-C, Table S2). In addition, familiar observers that received the anti-orexin antibody had less allo-grooming and allo-licking toward surgery mice and spent more time in the light zone of the light and dark box than familiar observers that received heat-inactivated anti-orexin antibody (Figures 8B-C). These results suggest a role of orexin receptor containing PVT neurons in the consolation and anxious behaviors of familiar observers.

## Discussions

Our results showed increased allo-groom of non-surgery mice toward surgery mice that had presentations of pain and distress. This behavior was higher in familiar observers than that in unfamiliar observers. However, the resident mice had reduced allo-grooming toward non-surgery intruders. Also, familiar observers had increased anxious behavior after interacting with surgery mice but not with non-surgery intruders. Surgery mice also had increased anxious behavior, which was attenuated by the interaction with familiar observers. These results suggest that familiar observers display consolation toward surgery mice because these behaviors of familiar observers are selective and pro-social and have a social buffering effect on surgery mice 3. Our results also suggest that the consolation of observers toward surgery mice was empathy-based because the consolation has the features of emotional contagion and self-regulation 3. These results support the establishment of a mouse model of consolation toward the sick. In addition to the emotional contagion (anxious behaviors between surgery mice and observers) and familiarity bias (more allo-grooming from familiar observers than that from unfamiliar observers toward surgery mice), our model showed physiological state-matching (such as IL-6 increase in the brain of surgery mice and familiar observers) and self-other differentiation. The evidence for self-other differentiation include decreased allo-grooming of resident mice toward non-surgery intruders, increased allo-grooming of observers toward surgery mice, lack of allo-grooming in surgery mice and no change in self-grooming in surgery mice and familiar observers. These results indicate that all-grooming from observers toward surgery mice is not a typical stress-related behavior but a specific consolation behavior toward the sick.

PVT receives inputs from many brain regions including prefrontal cortex and lateral hypothalamic area 26. These areas provide orexinergic, glutamatergic and GABAergic modulation of PVT 19, 32, 33. Neurons in PVT are glutamatergic neurons that send output to various brain regions including medial prefrontal cortex, Acb, amygdala and insular cortex, regions that are involved in emotion regulation 26, 34. PVT has been shown to be involved in wakefulness, learning and food intake behavior 19, 27, 28. PVT is also involved in emotional behavior and fear learning and memory 35, 36. Our results suggest additional neurobiological functions for PVT: controlling the consolation behavior of familiar observers toward the sick and anxious behavior after interacting with the sick. In supporting this role, PVT in familiar observers was activated after interaction with surgery mice but this activation did not occur in resident mice that interacted with non-surgery intruders. PVT neuron damage by ibotenic acid, inhibiting PVT neurons by a chemogenetic approach or blocking the orexin signaling of PVT neurons by an anti-orexin antibody attenuated the consolation of familiar observers toward surgery mice and anxious behavior of the observers after interacting with the sick (Figure 8D).

Our results showed that PVT neurons were activated in surgery mice, familiar observers, unfamiliar observers and separated observers. Similar to familiar observers, unfamiliar observers had consolation toward surgery mice and increased anxious behavior after interacting with surgery mice. The consolation behavior could not be measured in separated observers due to experimental setup. However, surgery mice did not have allo-grooming toward familiar observers or other surgery mice but had increased anxious behavior. Thus, the activation of PVT neurons in surgery mice cannot explain the lack of consolation of these mice toward others. The lack of consolation in surgery mice is conceivable because these mice were sick and suffered from pain and distress. Consistent with this possibility, physically stressed Mandarin voles have reduced consolation toward their social defeated partners 8.

Our results showed that blocking orexin signaling in PVT attenuated consolation and anxious behaviors in familiar observers, suggesting a role of orexin signaling in these behaviors. Consistent with our findings, orexin has been found to regulate anxiety and adaptation to stress 37, 38. Orexin signaling may be involved in consolation of Mandarin voles toward socially defeated partners 8, 39 Orexinergic modulation of PVT neurons is reported from lateral hypothalamic area 19, 34. Our findings imply a role of lateral hypothalamic area in the consolation and anxious behaviors of familiar observers.

The Russian-doll model has been used to arrange the forms of empathy into layers. Emotional contagion and motor mimicry, such as empathic contagious pain, yawning and contagious itching, has been shown in small animals including mice and is the innermost layer of empathy. The next layer is consolation that requires self-regulation 3. Very limited studies have investigated consolation behavior in small animals. Consolation of cage-mates toward others that were stressed by foot shocks was observed in highly social prairie vole but was not observed in meadow voles raised in breeding colony of laboratory. This consolation was associated with activation of anterior cingulate cortex and was oxytocin-dependent 4. Consolation toward others that had pain induced by injection of bee venom into hind paw was observed in mice and rats. Neurobiological mechanisms for this consolation have not been reported 9, 40. Although consolation behavior in humans is common, neurobiology for this behavior is difficult to study because consolation is a form or component of altruism, the outmost layer of empathy that often requires the involvement of executive function 3. Imaging studies in humans have shown that sympathy (consolation is a behavioral presentation of sympathy) is associated with activation of middle insula and prefrontal cortex 41, 42. The degree of altruism is associated with the level of coupling between amygdala and midbrain periaqueductal grey 43. However, investigation on neural circuit and molecular mechanisms for the higher forms of empathy including consolation and altruism are at infant stage. Our study suggests a role of orexin signaling in the PVT neurons in the consolation of familiar observers toward the sick surgery mice. These consolation behaviors may have beneficial effects on the sick because surgery mice after being interacted with familiar observers had less anxious behavior than surgery mice that stayed with other surgery mice. However, our results did not show a decreased pain and distress presentation by the consolation.

IL-6 was measured as an indicator of inflammation and stress 23, 44. Similar to our previous findings 17, IL-6 levels in the blood peaked quickly after surgery and this increase was reduced afterwards, suggesting that systemic inflammation decreases with time after surgery. Surgery mice also had increased IL-6 in the cerebral cortex. Although IL-6 in the hippocampus was not increased within 24 h after surgery in this study, our previous studies have shown increased IL-6 and inflammation in mouse hippocampus after surgery 44. Interestingly, familiar observers did not have an increase in blood IL-6 but had an increased IL-6 level in the brain, suggesting an effect via sensory input other than vision or smell, into the brain because separated observers did not have increased IL-6 in the brain. The increased IL-6 in the brain of familiar observers may not require the activation of PVT because PVT neuron damage by ibotenic acid did not affect the increase of IL-6 in the brain. The increased IL-6 in the brain may not be needed for PVT activation in the mice because IL-6 increase took 6 h and PVT neuronal activation was present 1 h after the interaction of familiar observers with surgery mice. Thus, the increase of IL-6 may not play a role in the consolation and anxious behaviors of familiar observers. However, the increased IL-6 in the familiar observers provides another line of evidence that the interaction with the sick induces brain changes, in addition to the consolation and anxious behavior, as we have shown previously 45.

Our study has limitations. As a treatment control group, we injected PBS (for ibotenic acid experiments) or heat-inactivated orexin antibody (for orexin antibody experiments) into PVT of familiar observers. The comparison between these treatment control groups with familiar observers without any injection in the performance of light and dark box tests was not different. However, the comparison between familiar observers receiving orexin antibody and familiar observers receiving heat-inactivated orexin antibody in the light zone time was different. Familiar observers received head-inactivated antibody remained spending more time in the grey and black zones than control mice (P = 0.008). It may not be appropriate to label this difference in figure 8C because there are two variables in familiar observers (familiar observer feature and injection to PVT) when compared with control mice. Together, these results suggest the role of orexin signaling in PVT in anxious behavior of familiar observers. However, the comparison of familiar observers receiving PBS injection and control mice in the performance of dark and light box was not different. The reasons for the less clear-cut evidence introduced by the results of familiar mice receiving PBS for PVT involvement in anxious behavior are not clear. However, injecting PBS into PVT may cause injury to the neurons in it, which may induce an effect similar to that of ibotenic acid. Another potential contributing factor is that the difference (effect size) between controls and familiar observers in the performance in the light and dark box tests and the changes caused by manipulations on PVT in this performance were smaller than those of allo-grooming and allo-licking. Nevertheless, despite of the less clear-cut evidence from the set of ibotenic acid experiment, the results from experiments using chemogenetic approach and anti-orexin antibody suggest the involvement of PVT in the anxious behavior of familiar observers after the interactions with surgery mice because inhibiting PVT neurons or orexin signaling in these neurons reduced anxious behavior of familiar observers compared with familiar observers without any treatment or familiar observers that received viral and vehicle injection or injection of heat-inactivated anti-orexin antibody. Another limitation of this study is that we have not clearly determined brain regions downstream of PVT for the consolation and anxious behaviors of familiar observers. We showed that neurons in PVT projected to Acb. Acb of familiar observers was activated and this activation was blocked by ibotenic acid-induced PVT neuronal injury. These results, along with previous finding that Acb is involved in anxious behavior 46, would suggest a role of Acb in the consolation and/or anxious behaviors of familiar observers. Our results also showed activation of insular cortex of familiar observers after interactions with surgery. Since insular cortex receives input from PVT and is involved in empathy 26, 47, insular cortex may be another brain structure downstream of PVT for consolation and anxious behaviors of familiar observers. The third limitation is that it is not known how PVT in familiar observers was activated. Social interaction between mice reduces PVT neuronal activation detected by a calcium sensor 48. This effect appears to be very quick (in seconds) but its duration is not known. Our results did not show a difference in c-Fos expression in PVT between surgery mice and surgery mice with familiar observers at the end of 1-h interaction. It is not known whether the lack of interaction between mice contributes to the activation of PVT in separated observers. Since parabrachial nucleus is a hub for pain and aversion 29 and sends projections to PVT, it is possible that parabrachial nucleus is activated, which then activates PVT in surgery mice. Our results show significant activation of parabrachial nucleus at 1 h after the surgery, which supports the role of parabrachial nucleus in the activation of PVT. Finally, we determined the role of PVT activation in the consolation and anxious behaviors of familiar observers but did not examine the role of PVT activation in the behavioral changes of surgery mice, unfamiliar observers and separated observers because familiar observers are the focus of our interest and may be the largest group for surgical patients. The role of PVT in consolation and anxious behavior of other observers and anxious behavior of surgery can be inferred from our findings but required additional experiments to confirm.

Together, our results provide initial evidence for the consolation behavior toward others with surgery in mice and the involvement of orexin neurons in PVT in both consolation and anxious behaviors in familiar observers (Figure 8D). Each year, more than 50 million patients have surgery in the United States 2. The neurobiological mechanisms for the consolation and anxious behaviors of their relatives and friends of this huge number of patients have not been reported. Our study may serve as a starting point to understand the mechanisms.

## Supplementary Material

Supplementary figures and tables.
