# Supplementary material for: Paraventricular thalamic nucleus plays a critical role in consolation and anxious behaviors of familiar observers exposed to surgery mice
Source: Theranostics. 2021 Feb 6;11(8):3813–29. doi: 10.7150/thno.45690 (PMC7914349; doi:10.7150/thno.45690)
Supplement: Supplementary file 1 — Supplementary figures and tables. [file thnov11p3813s1.pdf]

Figure legend for supplemental figures

Familiar observers/Surgery mice

	Ratio	P value
HIPPO	1.24	0.2189
ZI	1.06	0.8191
VMH	0.98	0.9210
Acb	0.87	0.2534
PVT	0.86	0.3801
PFC	0.73	0.0409
Pir	0.59	0.0557
DM	0.54	0.0086
XI	0.47	0.0022
IMD	0.20	0.0001
BMA	0.00	0.0001
CM	0.00	0.0001

Figure S1. Heat map of the comparisons between familiar observers and surgery mice.

Table S1. KEY RESOURCES

REAGENT or RESOURCE	SOURCE	IDENTIFIER
**Antibodies**		
Rabbit polyclonal to c-Fos	Abcam	Cat#ab190289
Mouse monoclonal [2H2] to c-Fos	Abcam	Cat#ab208942
Rabbit orexin receptor 1+2 polyclonal antibody	Bioss antibodies	Cat#bs-1095R
Anti-NeuN Antibody, clone A60	Sigma-Aldrich	Cat#MAB377
Rabbit polyclonal to Orexin A	Abcam	Cat#ab6214
Donkey anti-Rabbit Alexa Fluor 488	Life Technologies	Cat#A21206
Donkey anti-Mouse Alexa Fluor 488	Invitrogen	Cat#A21202
Donkey anti-Rabbit Alexa Fluor 594	Invitrogen	Cat#A21207
Donkey anti-Mouse Alexa Fluor 594	Invitrogen	Cat#A21203
Hochest	Thermo Scientific	Cat#RC 21732420
**Viruses**		
pAAV2-hSyn-hM4D(Gi)-mCherry	Addgene	Cat#50475-AAV2
pAAV2-hSyn-mCherry	Addgene	Cat#114472-AAV2
pAAV2-hSyn-EGFP	Addgene	Cat#50465-AAV2
**Drugs**		
DREADD Agonist 21	Abcam	Cat#ab235545
Ibotenic acid	Abcam	Cat#ab120041
**Critical Commercial Assays**		
Mouse IL-6 Immunoassay	R&D systems	Cat#M600B
**Chemicals**		
10× RIPA Buffer	Thermo Scientific	Cat#89901
Ketamine	Zoetis	N/A

Table S2. Statistical values of ANOVAs

**Data location in the figures**	F or H values	P values
Figure 1C (Grey + black zones)	F(2,36) = 7.609	0.002
Figure 1C (light zones)	F(2,360 = 13.156	< 0.001
Figure 2E	H = 4.550 (df = 2) on rank	0.103
Figure 4B	F(3,20) = 11.505	< 0.001
Figure 4D	F(3,22) = 14.383	< 0.001
Figure 4E	F(3,25) = 5.962	0.003
Figure 4G	F(2,12) = 19.517	< 0.001
Figure 4I	F(4,24) = 6.188	0.001
Figure 5A at 2 h after interaction	F(4,21) = 68.619	< 0.001
Figure 5A at 6 h after interaction	F(4,21) = 41.494	< 0.001
Figure 5A at 24 h after interaction	F(4, 21) = 6.065	0.002
Figure 5B at 2 h after interaction	F(4,21) = 1.166	0.354
Figure 5B at 6 h after interaction	F(4, 21) = 5.688	0.003
Figure 5B at 24 h after interaction	F(4,21) = 4.501	0.009
Figure 5C at 2 h after interaction	F(4,21) = 2.642	0.062
Figure 5C at 6 h after interaction	F(4,21) = 13.857	< 0.001
Figure 5C at 24 h after interaction	F(4,21) = 17.475	< 0.001
Figure 5D Serum	F(2,15) = 2.516	0.114
Figure 5D Hippocampus	F(2,21) = 3.577	0.046
Figure 5D Cerebral cortex	F(2,19) = 6.515	0.007
Figure 6D Allo-grooming	F(2,34) = 28.913	< 0.001
Figure 6D Allo-licking	F(2,34) = 6.123	0.005
Figure 6D Allo-grooming + all-licking	F(2, 34) = 32.045	< 0.001
Figure 6E Grey + black zone	F(3,45) = 4.267	0.010
Figure 6E Light zone	F(3,45) = 4.267	0.010
Figure 7B Allo-grooming	H = 11.901 (df = 3) on rank	0.008
Figure 7B Allo-licking	H = 9.275 (df = 3) on rank	0.026
Figure 7B Allo-grooming + all-licking	H = 12.711 (df = 3) on rank	0.005
Figure 7C Grey + black zone	F(4,56) = 2.942	0.028
Figure 7C Light zone	F(4,56) = 3.425	0.014
Figure 8B Allo-grooming	F(2,36) = 5.847	0.006
Figure 8B Allo-licking	F(2,36) = 4.197	0.023
Figure 8B Allo-grooming + all-licking	F(2,36) = 8.771	< 0.001
Figure 8C Grey + black zone	F(3,50) = 4.283	0.009
Figure 8C Light zone	F(3,50) = 5.435	0.003
